# Epitaxial Non *c*-Axis *Twin-Free* Bi_2_Sr_2_CaCu_2_O_8+δ_ Thin Films for Future THz Devices

**DOI:** 10.3390/ma12071124

**Published:** 2019-04-05

**Authors:** Kazuhiro Endo, Shunichi Arisawa, Petre Badica

**Affiliations:** 1 Kanazawa Institute of Technology, Hakusan, Ishikawa 924-0838, Japan; 2 National Institute of Materials Science, Tsukuba, Ibaraki 305-0047, Japan; arisawa.shunichi@nims.go.jp; 3 National Institute of Materials Physics, street Atomistilor 405 A, 077125 Magurele, Romania

**Keywords:** Bi_2_Sr_2_Ca_2_CuO_8+δ_, non *c*-axis epitaxial thin films, X-ray diffraction, twins

## Abstract

Thin films of (117) Bi_2_Sr_2_Ca_2_CuO_8+δ_ (Bi-2212) were grown by Molecular Organic Chemical Vapor Deposition (MOCVD) on (110) SrTiO_3_ and (110) LaAlO_3_ substrates. Substrates were vicinal with off angles up to 20°. Films are 3D epitaxial and X-ray diffraction *φ*-*ψ* scans demonstrate that, while the films grown on a flat substrate are composed of twinned grains, the films on vicinal substrate are *twin-free*. A higher quality is obtained if growth is performed at two temperatures: Growth starts at 550–600 °C and continues at 700–750 °C. The twin-free film grown by the two-temperature method shows a zero-resistance critical temperature of 37 and 32 K when the measuring current is applied in-plane parallel and perpendicular to [001] direction of the substrate. Twin-free non *c*-axis thin films are promising for fabrication of novel planar THz devices.

## 1. Introduction

Over the last few years, interest in THz devices has surged due to possible applications in cancer diagnosis, food inspection, explosive detection, electronics and communications, among others. Emission and detection of THz waves are achieved by different methods such as quantum cascade lasers [1], resonance tunneling diode oscillators [2,3], and the use of intrinsic Josephson junctions (IJJ) [4]. The IJJ works at low temperatures, but it has the advantage of generating tunable, coherent, monochromatic and continuous THz waves. Small and high performance solid state THz devices are expected to be fabricated by using IJJ [4,5,6]. In this regard, highly anisotropic layered high temperature superconductors (HTS) such as cuprates are key materials. The typical tetragonal or orthorhombic crystal lattice of HTS cuprates can be viewed as a stack in the *c*-axis direction of Cu-O superconducting (S) blocks separated by non-superconducting blocks (insulating, I). If a current is applied along the *c*-axis, it will pass though the SIS structure by Josephson tunneling effect. The intrinsic Josephson effect was reported for both YBa_2_Cu_3_O_7_ and Bi-based superconductors [7,8,9,10,11]. However, the Bi-based superconductor is considered more appropriate for fabrication of IJJ devices given that it has a higher anisotropy parameter. In particular, phases in the Bi-Sr-Ca-Cu-O (BSCCO) HTS system such as Bi_2_Sr_2_CaCu_2_O_8_ (B2212) and Bi_2_Sr_2_Ca_2_Cu_3_O_10_ (Bi2223) are ideal candidates for IJJ-THz device fabrication.

The IJJ-THz devices show some drawbacks. Among them we note the limitations induced by perpendicular emission of THz waves on *c*-axis. Namely, this means that a *c*-axis epitaxial film or bulk single crystal will emit or detect THz waves only through its lateral limited surface. To have an array of coherent emitters or detectors and to expand the number of IJJ per surface, one idea would be to grow thicker *c*-axis thin films or bulk crystals. As it is well known, a larger thickness leads to a lower quality of a film: High uniformity is not achieved and the THz device quality will be suppressed. This analysis also explains why chemical coating techniques, although cheap and suitable for growth of thick films, but with a relatively low level of processing control are not expected to generate films with required top quality for THz devices.

**Figure 1:**
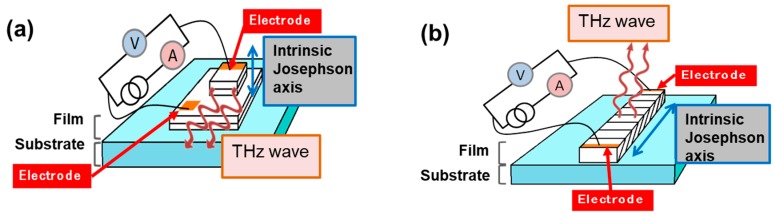
Schematic drawing of intrinsic Josephson junction devices for THz emission: (**a**) Mesa-type using *c*-axis high temperature superconductors (HTS) oriented film; (**b**) Planar-type using non *c*-axis HTS oriented film.

Our proposal is to grow by MOCVD and to use non *c*-axis epitaxial thin films for IJJ-THz device fabrication. On one hand, this is expected to enhance the emission power and detection capacity due to a higher density of active IJJ, and on the other it may promote design of new planar devices (Figure 1). Thin films are also thought to be more useful than bulk crystals for integration purposes and, in fact, a non *c*-axis film can be viewed as an organized structure of many nano or micro scale crystals with the same orientation and quality. It is also worthy to mention that integration of thin films with different orientations into heterostructures [12,13,14,15,16] promotes formation of novel synergetic effects at interfaces that are useful for the development of new devices.

Films orientation is controlled by selecting appropriate substrates with a suitable film-substrate lattice matching relationship. In our previous works we have grown *c*-axis and non *c*-axis HTS films on different substrates [17]. The layer-by-layer growth of (117) Bi2212 films by Molecular Organic Chemical Vapor Deposition (MOCVD) on flat substrates was demonstrated to generate twinned films. Twins are not desirable for IJJ-THz applications. To suppress formation of the twins, in this work, MOCVD growth of (117) Bi2212 thin films was performed on vicinal substrates employing a step-flow growth mechanism. X-ray diffraction advanced characterization using 2*θ*-*θ* and *φ*-*ψ* scans shows formation of twin-free 3D epitaxial thin films and results are compared with twinned films on flat substrates. Films were grown by one-temperature and two-temperatures (template) approaches. In the two-temperature approach, the film is grown first at a lower substrate temperature (550–600 °C) and the process continues at a higher temperature (700–750 °C). Films grown by the two-temperature method show a higher quality. A film grown by the two-temperature method on a vicinal substrate exhibit a zero-resistance critical temperature of 37 and 32 K when the measuring current is applied in-plane, parallel and perpendicular. Scanning electron microscopy (SEM) and atomic force microscopy (AFM) investigations were used to reveal the morphology and to correlate with orientation-results obtained by X-ray diffraction (XRD).

## 2. Materials and Methods

Non *c*-axis thin films of (117) Bi-2212 were grown by metalorganic chemical vapor deposition (MOCVD). Source materials (Toshima Manufacturing Co., Ltd., Higashimatsuyama, Japan) were Bi(o-tolyl)_3_ and M(DPM)_2_ with M = Sr, Ca and Cu. DPM is the abbreviation for dipivaloylmethane ligand. Vapors were supplied to the reaction chamber by a stream of Ar-carrier gas with the following flow rates: 100, 300, 300 and 70 mL/min for Bi, Sr, Ca and Cu, respectively. The substrate was inductively heated at 500–800 °C. In the one-temperature approach the heating temperature was maintained for a deposition time of 25–110 h. In the case of the two-temperature route, deposition of the films was performed at a temperature of 500–600 °C for 50–70 h and further at a higher temperature of 700–800 °C for 60–70 h. Oxygen was introduced in the reaction tube with a partial pressure of 2.6 kPa and a flow rate of 640 mL/min. The total pressure in the chamber was 6.7 kPa. More details on the equipment are presented in refs. [18,19]. Films were grown on flat and vicinal (110) SrTiO_3_ (STO) and (110) LaAlO_3_ (LAO) single crystal substrates produced by Furuuchi Chemical Co., Tokyo, Japan. The off (miscut) angle of the as-received flat substrates was measured by AFM to be less than 0.3°. Vicinal substrates were with an off angle (|α|) of 10 or 20°. The substrate was selected to match the most probable theoretical in-plane relationship between the lattices of the substrate and the film. Namely, the (117) plane of the Bi-2212 matches the (110) plane of the substrate [16].

The thickness of the films was determined considering the mass estimated from measurements of inductively coupled plasma spectroscopy (ICP-AES, SPS 7700, Hitachi High-Tech Co., Tokyo, Japan) and the theoretical density of the Bi-2212 phase (6.6 g/cm^3^). Films were further characterized only if their cation composition determined by ICP was close to 2212. Composition of the films presented in this work is Bi_1.9–2.18_Sr_1.82–2.02_Ca_1.9–2.05_Cu_1.9–2.14_O_x_.

XRD measurements were performed on Ultima (Rigaku Co., Tokyo, Japan) and X’Pert MRD (Malver-Panalytical, Malvern, UK) diffractometers (CuKα radiation) and they were of 2*θ*-*θ* and of *φ*-*ψ* type, respectively. The *φ*-*ψ* scans were taken for 2θ = 29.048° corresponding to (0010) plane of Bi-2212. For a fixed *ψ* within (−85°, 85°), the *φ*-scan was measured. Other details of the *φ*-*ψ* scan methodology are described elsewhere [17].

The morphology of the non *c*-axis films was observed by AFM and SEM using the microscopes SPA 400 (Hitachi High-Tech Co., Tokyo, Japan) and S-3400N (Hitachi High-Tech Co.), respectively.

Resistivity vs. temperature of the thin films was measured by the standard four-probe technique. The measuring current *I* of 5 mA was applied in the plane of the substrate parallel and perpendicular to the [001] direction. Electrical contacts were made by silver paste.

## 3. Results and Discussion

### 3.1. Thin Films Grown on Flat Substrates

**Figure 2:**
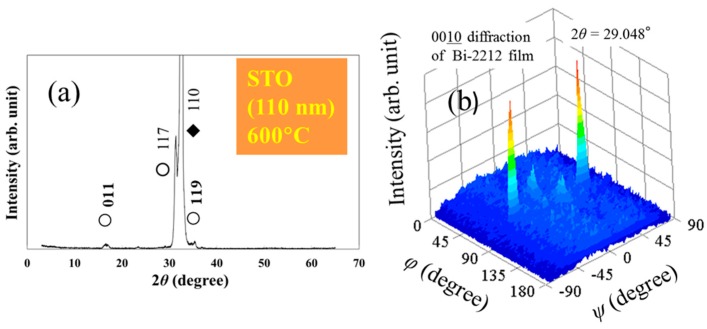
(**a**) X-ray diffraction patterns (2*θ*-*θ*) and (**b**) *φ*-*ψ* scans ((0010) plane, 2*θ* = 29.048°) for the non *c*-axis Bi-2212 thin film grown at 600 °C on flat (110) SrTiO_3_ substrate. Phases are: ○ Bi-2212, ♦ substrate. Positions (*ϕ, ψ*) of the peaks in (**b**) are: (90°, 45°) and (90°, −45°).

**Figure 3:**
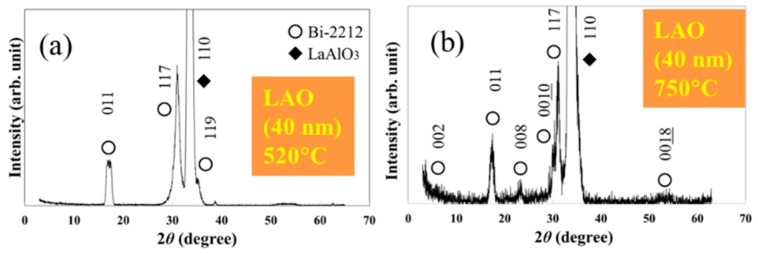
XRD patterns of non c-axis Bi2212 films grown on flat (110) LAO substrate at (**a**) 520 and (**b**) 750 °C (orientations and thickness are also indicated).

According to 2*θ*-*θ* XRD patterns taken on the non *c*-axis Bi-2212 thin films grown by one-temperature route at temperatures between 520–750 °C on flat STO or LAO substrates (Figure 2a and Figure 3), the main orientation is (117). The impurity orientations are (011) and (119) at temperatures below 700 °C and (011) and (001) above it. High temperatures (e.g., 750 °C in Figure 3b) promote formation of more grains with impurity orientations than at lower temperatures (Figure 3) and these grains are mostly with (001) orientation. Impurity orientations (110) and (119) show a relatively convenient matching relationship with the (110) plane of the substrate, as explained in ref. [17]. The presence of the (001) impurity orientation, not matching the substrate, is perhaps a consequence of the high mobility of the adatoms at high temperatures and of the crystal chemistry of the Bi-2212 phase. Bi-2212 has strong chemical bonds in the ab-plane and weak van der Waals bonds out of plane (in the *c-*axis direction). Therefore, strong bonds condition is equivalent with a preferentially lower local crystallization energy for the *ab*-plane of Bi-2212 so that it can form on a substrate surface even if its lattice matching with substrate is not convenient. The process is aided by specific depositions conditions such as high temperatures in our case. To avoid formation of a large amount of impurity orientations, low or intermediate temperatures of growth would be recommended. Good results in this respect are obtained for a growth temperature of 600 °C (Figure 2a).

When growth conditions are fixed, less impurity orientations are found for the non *c*-axis films grown on LAO than for the films deposited on STO. This might be due to lower values of the mismatch coefficients for LAO than for STO; along [001] and [110] directions of the (110) substrate, the mismatch values are 0.98% and 8.95% for LAO [17] and 3.88% and 11.63% for STO.

The *φ*-*ψ* scans show comparable results for films on STO (Figure 2b) and LAO [17] grown at 600 °C. There are two peaks located at approximately (*φ* = 90°, *ψ* = ±45°). Growth mechanism is similar for both substrates. The values of *ψ* = ±45° are close to theoretical values of 41.03° for (117) and 48.23° for (119) orientations. Kuroda et al. [12] measured by Reflection High-Energy Electron Diffraction (RHEED) an angle of about 48° for a (11n) Bi-2212 thin film. As concluded in reference [17] our non *c*-axis films on flat substrates have the *c*-axis inclined under the experimental *ψ*-angles (defined in respect to the normal to the substrate surface). Therefore, two opposite and inclined growth fronts develop. The non *c*-axis film grows in the *c*-axis direction by a layer-by-layer mechanism through addition of *ab*-planes. This mechanism is as for the growth of the *c*-axis thin film except that for the *c*-axis film the *c*-axis is perpendicular to the substrate’s surface (*ψ* = 0°). When the two opposite inclined growing fronts merge, a span roof-like grain forms and it contains a twin boundary that is parallel to the (001) direction of the substrate.

**Figure 4:**
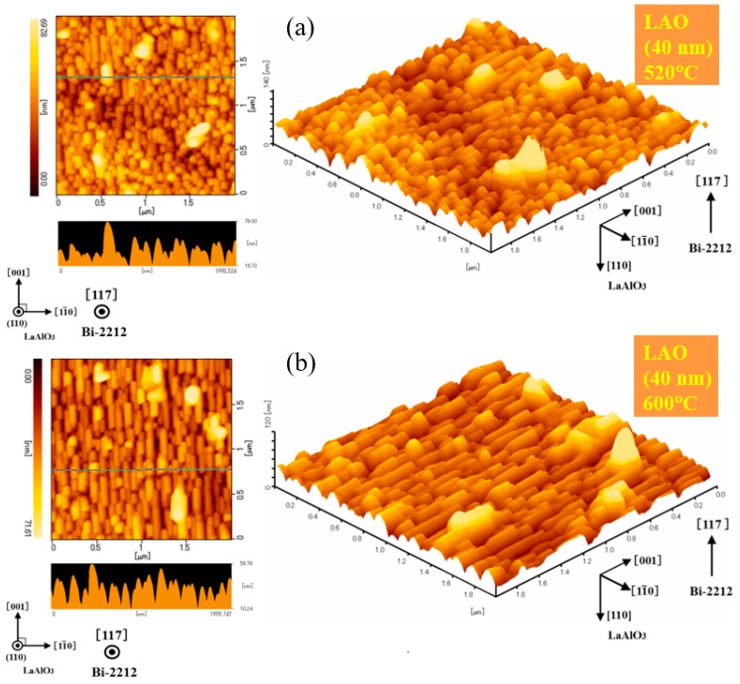
AFM 2D and 3D images and roughness profile for the non *c*-axis Bi-2212 thin film on flat (110) substrate grown at (**a**) 520, RMS = 9 nm and (**b**) 600°C, RMS = 12.06 nm.

**Figure 5:**
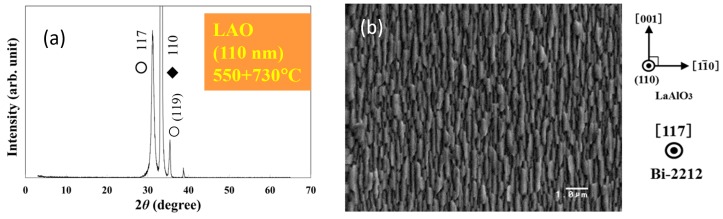

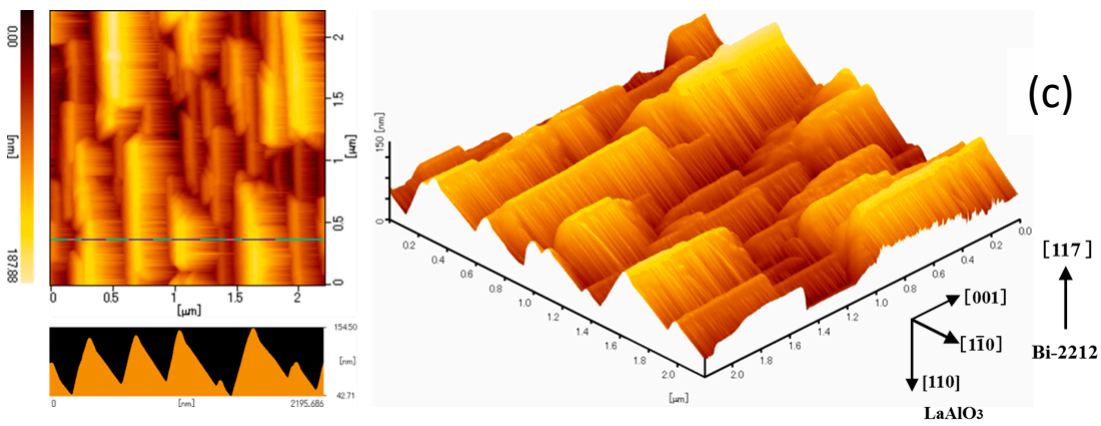
Non *c*-axis Bi-2212 thin film on flat (110) LaAlO_3_ substrate obtained by two-temperature growth: (**a**) XRD pattern, (**b**) SEM image and (**c**) AFM 2D and 3D images and roughness profile (RMS = 33.2 nm). Phases are: ○ Bi-2212, ♦ substrate.

The roof-like morphology is revealed by AFM images in Figure 4. These images support the idea that non *c*-axis films on flat substrates are 3D epitaxial. A better morphological uniformity is found when growth is performed at higher temperatures. For example, in Figure 4 one observes from the roughness profile lines that the shape of the grains in the Bi-2212 films grown at 600 °C is more uniform than in the film obtained at 520 °C. In fact, in the films fabricated at 520 °C there are grains without a clear roof-like shape (Figure 4a) and they show a spheroidal shape. Even in the film grown at 600 °C (Figure 4b) there are grains departing from the roof-like geometry. It is inferred that a higher crystal quality is for a higher growth temperature. Our observations suggest that temperatures above 600 °C are useful to grow films with uniform roof-like microstructure composed of grains with a higher crystal quality. However, we shall recall the result from the previous paragraphs where we have shown that higher growth temperatures promote formation of a larger amount of undesirable impurity orientations especially of the (001) orientation. To take advantage of the low and high temperature growth features, and to control and optimize the films quality, application of a two-temperature growth approach is proposed: The low temperature layer will act as a template for the high temperature layer. First, we grow the film at a temperature of 550–600 °C and deposition continues at a temperature of 700–730 °C. Results for a film grown by two-temperature method on flat (110) LAO at 550 and 730 °C are presented in Figure 5. The uniformity of the roof-like grains in the Bi-2212 films is improved (see Figure 5c—roughness profile and compare with Figure 4), while grains with other geometries are not visible (Figure 5b,c—3D AFM image). XRD pattern (Figure 5a) shows the presence of the main (117) orientation and of the (119) impurity orientation. A relatively small unidentified peak occurs at 2*θ* = 38°. The most inconvenient orientation (001) for fabrication of the planar device proposed in Figure 1b is missing. An additional possible argument supporting the necessity of a two-temperature growth is based on our previous experience on the growth and characterization of non *c*-axis Bi-2223 thin films and on their growth similarities with those of the Bi-2212 films from this work. Films of Bi-2223 grown at higher temperatures had higher critical temperatures than for the films grown at lower temperature [16]. Extrapolation of the results from Bi-2223 to Bi-2212 requires further confirmation.

### 3.2. Thin Films (Twin-Free) Grown on Vicinal Substrates

**Figure 6:**
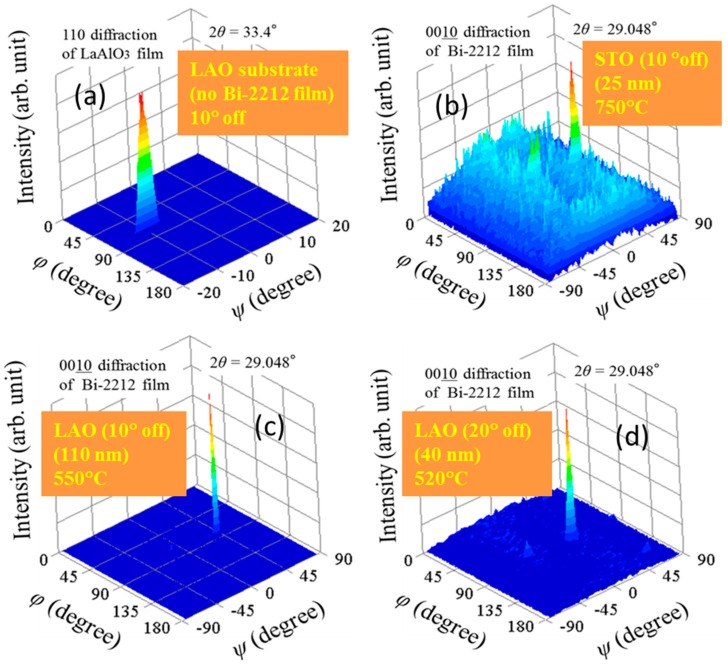
XRD *φ*-*ψ* scans for the (**a**) (110) LaAlO_3_ (LAO) substrate (110) plane, 2*θ* = 33.4°) and (**b**–**d**) non *c*-axis Bi-2212 thin films (0010) plane, 2*θ* = 29.048°) obtained for different growth temperatures on different vicinal substrates. Positions (*ϕ, ψ*) of the peaks in (**a**–**d**) are: (81°, −10°), (90°, 37°), (90°, 36°), and (91°, 25°), respectively.

**Figure 7:**
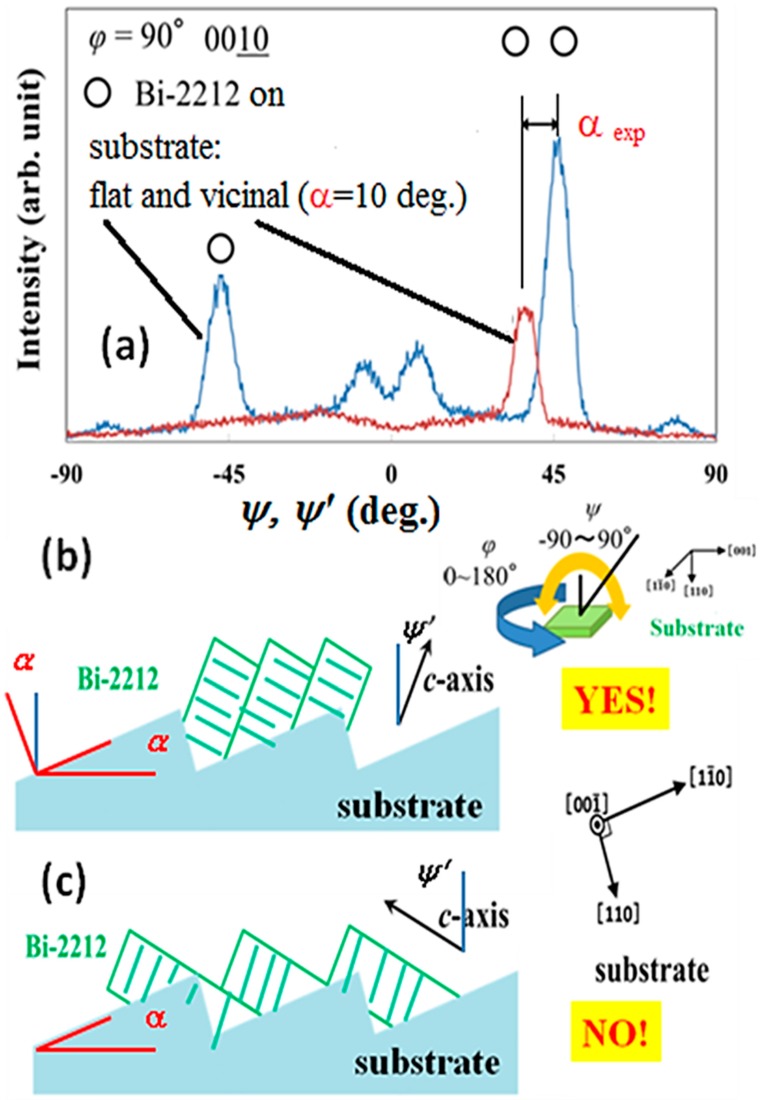
(**a**) XRD *ψ* and *ψ*’ scans for constant *φ* = 90° of the (0010) plane (2*θ* = 29.048°) on the non *c*-axis Bi-2212 thin films grown on flat and vicinal (off angle 10°) STO substrates, respectively; (**b**,**c**) Theoretical models for the step-flow growth mechanism that promotes twin-free growth. Results indicate that growth of the non *c*-axis Bi-2212 thin film takes place according to model from (**b**). Inset to (**b**) shows a schematic drawing of the *φ*-*ψ* measurement arrangement for the film grown on a flat substrate.

Films of non *c*-axis Bi-2212 grown on the vicinal substrates (|α| = 10 or 20°) show in the *φ*-*ψ* space ((0010) plane, 2*θ* = 29.048°) only one peak (Figure 6b,d). For a vicinal substrate, the *φ*-*ψ* scan (LAO, Figure 5a) indicates an off angle (*ψ*_substrate_ = *α*, Figure 7b) of about 10° in absolute value as expected and provided by the substrate supplier. The presence for the Bi-2212 non *c*-axis thin films on vicinal substrates of only one peak in the *φ*-*ψ* space (*φ* = 90° and 0< *ψ* < 45°) instead of two as for the flat substrates (compare Figure 6b–d and Figure 2b,d; see also Figure 7a) indicates on preferential directional growth. Namely, the two opposite merging growth fronts as for the flat substrates generating twinned span roof-like grains do not occur and only one growth front develops. The explanation resides in the fact that for the flat substrate nucleation is random, while for the vicinal substrate it is at the edges of terraces formed on the surface of the vicinal substrate. The network of parallel terraces’ edges is a convenient nucleation site where the free energy is lower and situation resembles a template growth. Therefore, on the initial stages, growth on a vicinal substrate is by the step-flow growth mechanism, while on the later stages the 2D layer-by-layer growth mechanism in the (inclined) *c*-axis direction, as for the flat substrate, is active. The main positive outcome of using vicinal substrates is that twins do not form and this is an important result for future fabrication of IJJ-THz planar devices. Further details and aspects of the twin-free morphology and of the growth on the vicinal substrate are addressed in the next paragraphs.

Two models of growth (with one growth front) on a vicinal substrate are considered (Figure 7b,c). To easily follow our presentation we shall introduce the following convention: In Figure 7, the angle formed by *c*-axis with the normal to the substrate surface is *ψ* when the film is grown on the flat substrate (see Figure 7b inset) and *ψ’* is for a vicinal substrate (Figure 7b,c). As addressed in Section 3.1, for a flat substrate *ψ* is approximately ± 45 °. One also observes that *ψ’* = *ψ* − |α|. If |*ψ’*| is increasing, the model from Figure 7c is valid (*ψ’* = −45° − |α|) and if it is decreasing (*ψ’* = 45° − |α|) the growth is according to the model from Figure 7b. Experimental XRD results of *φ*-*ψ* scan (*φ* = 90°) from Figure 7a show that |*ψ’*| is decreasing from about 45° (flat substrate) to about 35° (vicinal substrate). Hence, |α_exp_| is about 10° and matches the value for the substrate |*α*| = 10°. In conclusion XRD characterization demonstrates that the model from Figure 7b describes growth of our twin-free films on vicinal substrates.

**Figure 8:**
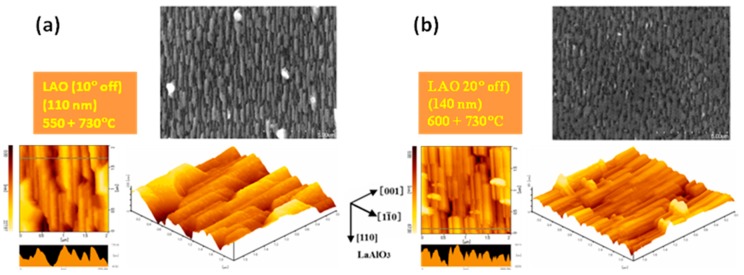
SEM and AFM images for non *c*-axis Bi-2212 thin films grown by the two-temperature method on vicinal (110) LAO substrates, (**a**) RMS = 38.46 nm, (**b**) RMS = 10.48 nm).

Although the twins do not form, the morphology of the grains from the non *c*-axis thin films of Bi-2212 on vicinal substrates resembles the span roof-like shape for the flat substrates (Figure 8). When compared to the film on the flat substrate (Figure 5), use of the vicinal substrate (|α| = 10°) in the two-temperature growth approach for constant growth conditions (550 + 730 °C) resulted in the decrease of the grains width (Figure 8a). A higher uniformity and a smaller roughness are obtained for a vicinal substrate with a larger off angle (|α| = 20°) (Figure 8b) and for a higher growth temperature in the first step of growth (550 + 730 °C) (compare Figure 8b with a). These improved features are necessary for fabrication of IJJ-THz devices, and have also a positive practical meaning if integration is required. The result can be understood considering the smaller width of the terraces and their higher density (suppressing random nucleation) for a vicinal substrate with a higher |α|. As in the case of the flat substrates, films on vicinal substrates according to AFM and SEM images (Figure 8) are aligned in-plane. Considering also structural data, we conclude that our films on vicinal substrates are twin-free 3D epitaxial thin films.

**Figure 9:**
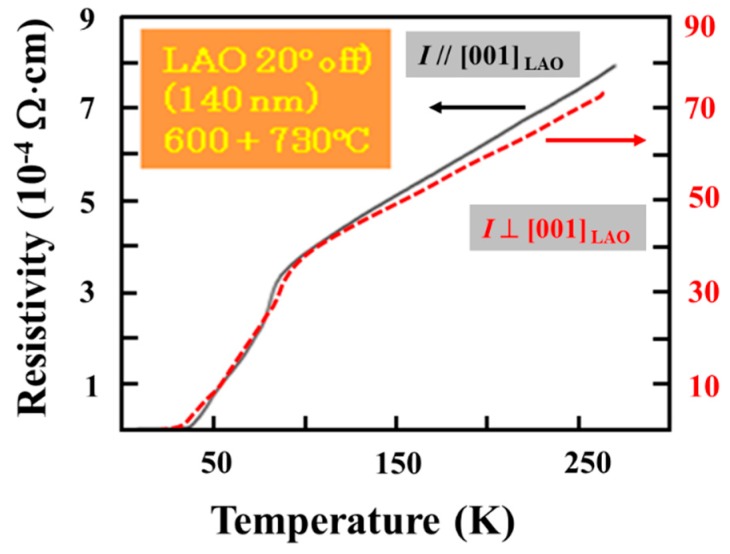
Resistivity vs. temperature measured on the film from Figure 8b when measuring current *I* is applied parallel (full line) and perpendicular (dashed line) to the [001] direction of the substrate.

Curves of the in-plane resistivity vs. temperature R(T) measured on the film from Figure 8b grown on the vicinal substrate by the two-temperature approach are presented in Figure 9. Zero resistivity critical temperature *T*_c(R__→0)_ is about 37 K and 32 K for the measuring current *I* applied parallel and perpendicular to the [001] direction of the substrate, respectively. For the first case the normal state resistivity (0.8 mΩ·cm), is about one order of magnitude lower than for the second case (7 mΩ⋅cm). Both R(T) curves show a wavy transition suggesting the presence of non-uniformities in the film. The specific orientation and morphology of the film provides an easy-path-condition for the current flow along the grains (*I*//[001]_Substrate_), i.e., the flow is (mainly) in the *ab*-plane. For the other case it generates a difficult path where the flow of the current has also a *c*-axis flow-component (*I* ⊥ [001]_Substrate_). The room temperature resistivity measured in the two directions, in plane, of a BSCCO non *c*-axis film (that is a mixture of (117) 2212 and (118) 2223 phases) grown on flat (110) STO in ref. [12] were 2 mΩ⋅cm and 4 mΩ⋅cm. The film had *T*_c(R__→0)_ = 48 K. Authors of reference [12] also noted that room temperature resistivity of a *c*-axis-oriented film grown under similar conditions on (100) STO was 3 and 6 times lower than for the non *c*-axis film on the flat (110)STO (considering the two directions of in plane measurement for the non *c*-axis film).

## 4. Conclusions

Thin films of Bi-2212 with a non *c*-axis orientation were grown by MOCVD using one-temperature or two-temperature routes on flat and vicinal (110) SrTiO_3_ and (110) LaAlO_3_ substrates. XRD, microscopy and R(T) characterization have shown that films are 3D epitaxial (117) Bi-2212 superconductor. By XRD *φ*-*ψ* scans it is demonstrated that, while the films on flat substrate are composed of twinned grains, the films on vicinal substrates are twin-free, and, thus, they show a higher quality and are expected to be useful for fabrication of IJJ-THz novel planar devices. In this work the zero-resistance critical temperature of the film grown by a two-temperature approach on a vicinal substrate with the off angle of 20° is 37/32 K for the measuring current applied in-plane parallel and perpendicular to [001] direction of the substrate.
